# Differences between criminal offender versus non-offender female patients with schizophrenia spectrum disorder: a retrospective cohort study

**DOI:** 10.1007/s00737-024-01477-7

**Published:** 2024-05-29

**Authors:** Lynn Jacobshagen, Lena Machetanz, Johannes Kirchebner

**Affiliations:** 1 Faculty of Medicine, University of Zurich, Zurich, Switzerland; 2 Department of Forensic Psychiatry, University Hospital of Psychiatry Zurich, Zurich, Switzerland

**Keywords:** Female, SSD, Schizophrenia, Psychiatry, Offence

## Abstract

The purpose of this study was to investigate the difference between offender female patients (OFS) and non-offender female patients (NOFS) with schizophrenia spectrum disorder (SSD).

The patients in this study were admitted to the university psychiatry in Zurich Switzerland between 1982 and 2016. Demography, psychopathology, comorbidity, and treatment differences were analyzed using binary statistics to compare 31 OFS and 29 matching NOFS with SSD. The Fisher‘s exact test was used for categorical data variables in small size samples and the Mann-Whitney-U-Test for nonparametric test variables, adjusted with the Benjamini and Hochberg method.

The results indicate that the NOFS were cognitively more impaired, they were more likely to have had antipsychotic drugs prescribed (NOFS; 100%, OFS: 71%, OR 1.41, 95% CI 1.13-1.77, *p=*0.022) and their medication compliance was higher (NOFS: 84.6%, OFS: 4.5%, OR 0.09, 95% CI 0.00-0.08, *p=*0.000). In contrast, the OFS had completed compulsory school less often and the were observed to be more often homeless and socially isolated (OFS: 72.4%, NOFS: 34.6%, OR 4.96, 95% CI 1.58-15.6, *p=*0.026), self-disorders (OFS: 51.6%, NOFS: 11.1%, OR 8.53, 95% CI 2.12-34.32, *p=*0.011), delusions (OFS: 96.8%, NOFS: 63%, OR 17.65, 95% CI 2.08-149.99, *p=*0.014) and substance use disorder (51.6%, OR 0.27, 95% CI 0.09-0.85, *p=*0.039). Clinicians treating female offender patients with SSD should focus more on the treatment for substance use disorder, medication and early recognition of the illness for preventative purposes.

## Introduction

Schizophrenia spectrum disorder (SSD) is associated with a higher risk of criminal (Hodgins et al. 2013) and violent (de Tribolet-Hardy and Habermeyer 2016; Fazel et al. 2009; Hodgins 2022) behavior, which explains the high percentage of offender patients with SSD in forensic psychiatric wards (de Tribolet-Hardy and Habermeyer 2016, approximately 50-60%). About 87 - 95% of the criminal offender patients in European forensic psychiatry are male (Hodgins 2022; Landgraf et al. 2013), which could explain why the majority of studies until now have concentrated on men when comparing offender vs. non-offender patients with SSD. However, women with SSD, compared to the men’s group, are at higher risk for violent offending (Hodgins 2022; Wang et al. 2017) as previous studies have shown. Fewer Women with SSD commit crimes than men with SSD, as seen in the general public as well, but SSD seems to influence aggressive behavior in women more strongly than in men (Hodgins 2022). How do female offender patients with SSD (OFS) differ from female non-offender patients with SSD (NOFS)?

Our study focused on OFS, their demography, social status, history, course of illness and their treatment possibilities, as scientific attention on female offender patients with SSD has been limited. Thus far, to our knowledge there is currently (January 2023) only one other paper that compares offender to non-offender female patients suffering from SSD (Landgraf et al. 2013), which can be explained by the extreme minority of women who are imprisoned.

Based on the knowledge about male patients with SSD criminal behavior development (further explained in section "Measures"), we hypothesized that female patients with SSD would show similar traits. This study’s object was to test the following hypothesis regarding those factors before their admission: OFS more likely live in social isolation, show a more aggressive behavior pattern towards others and suffer from more SSD specific symptoms than NOFS.

## Methods

### Setting

The Centre for Inpatient Forensic Therapy at the University Hospital of Psychiatry Zurich is the largest adult inpatient forensic psychiatric facility in German-speaking Switzerland. Offender patients are either referred for treatment of acute syndromes or for court-mandated therapy to reduce the risk of reoffending due to their underlying psychiatric disorder. The majority of offender patients are diagnosed with a disorder on the schizophrenia spectrum.

### Sample characteristics

As part of an overarching research project, we examined treatment records from patients admitted between 1982 and 2016, with the majority being recorded after the year 2000. From those records, we only included female patients diagnosed with schizophrenia spectrum disorder according to ICD-9 or ICD-10 (chapters F20.0 to F25.9; International Statistical Classification of Diseases and Related Health Problems. 3 2004; Slee 1978). In total, the OFS was comprised of 31 cases. There were no further exclusion criteria. All of the forensic female patients from our records were included in this study. This is because female offender patients represent a noticeable minority in the offender population and thus, in order to obtain strong statistical results, we included every female forensic patient. And also, we aimed to create results, which would support a foundation of knowledge about this neglected population, and while subgroup analyses e.g. of female offenders with certain comorbidities would have been interesting, they would not have been statistically feasible given the small sample. Offenses leading to the referenced forensic psychiatric hospitalization included violent crimes: (attempted) homicide, assault, violent offenses against sexual integrity, robbery, and arson - as well as non-violent crimes such as: threat and coercion, property crime without violence, criminal damage, traffic offenses, drug offenses, and illegal gun possession. The reason for inclusion of non-violent offenders was to reach our goal of comparing female offenders and non-offenders, regardless of the severity or type of their offense. In doing so, we attempted to cover the whole spectrum of criminal behavior, instead of narrowing it down to just aggressive acts.

Our comparison group consisted of 29 female non-offenders with SSD according to ICD-9 or ICD-10 (NOFS), who had been in general psychiatric inpatient treatment at another institution of the University Hospital of Psychiatry Zurich, the Centre for Integrative Psychiatry (International Statistical Classification of Diseases and Related Health Problems. 3 2004; Slee 1978). Apart from other specialized wards, this institution provides a sub-acute setting for psychotic disorder therapy, usually enduring 6 to 8 weeks. We chose to study this population for two reasons: first, just as in the forensic sample, the majority of patients suffered from a chronic, prolonged course of SSD. Second, most of the patients were referred with an established pharmacotherapy. The same is true for the forensic sample, where patients were usually primarily treated when being in custody (or other penitentiary settings), and thus already had an antipsychotic prescription upon admission.

### Measures

The hypotheses and the selection of variables are based on the first author’s preexisting knowledge of the development of criminal behavior among (nostly) male patients and on the papers by Barlati et al. 2022; Günther et al. 2021; Hodgins 2022; Landgraf et al. 2013; Wang et al. 2017, 2019. Further details about the variables are explained in the appendix. The literature research on "pubmed" was based on the following keywords: female, women, sex, gender, offender, offence, schizophrenia, SSD and forensic psychiatry.

### Extraction of data

Data from both the study and the comparison group was assessed as directed qualitative content analysis (Hsieh and Shannon 2005). Two experienced psychiatrists systematically reviewed the comprehensive patients’ records and conducted the data extraction by means of a modified rating protocol which was based on a set of categories proposed by (Seifert and Leygraf 1997). The case files included reports by various health care disciplines regarding the referenced institutionalizations, previous psychiatric hospitalizations, outpatient treatments, and, if applicable, police and court reports and testimonies. For estimation of inter-rater reliability, a random sub-sample of 10% of the cases was independently rated by a third health care professional. Cohen’s Kappa value (Brennan and Hays 1992) was 0.78, which can be regarded as substantial. All variables described in the appendix were rated as being present whenever case files explicitly stated their presence or included diagnostic results proving their existence. In order to estimate inter-rater reliability, a second trained independent rater encoded a random subsample of 10% of the cases previously rated by the first author. Cohen’s Kappa value was 0.78, which is substantial (Brennan and Hays 1992).

### Statistical Analysis

Statistical data analysis was conducted on SPSS version 23. All categorical data were described as number of subjects and percentages. Significant differences between OFS and NOFS were identified by means of Fisher’s exact test, since the sample was small (Freeman and Campbell 2007; Bower 2003). Association was measured by the odds ratio (OR) and 95% confidence intervals (CI). The continuous variable "age" was analyzed using the Mann-Whitney-U-test due to its non-normal distribution (Mann and Whitney 1947). All p-values adjusted for multiplicity were obtained by the Benjamini and Hochberg method ( Benjamini et al. 2001; Ferreira and Zwinderman 2006). All tests were two-tailed, and the significance level was defined as *p* < 0.05.

## Results

### Demography

Firstly, per Table 1, the two groups differed sociodemographically: OFS were significantly more often homeless at the time of the offence (OFS 23.3%, NOFS at admission 0%, OR=1.3, CI=1.07-1.59, *p=*0.035) and they significantly more often failed to have completed compulsory school (27.6%) NOFS (0%, OR=1.38, CI=1.1-1.73, *p=*0.031). However, there was no significant difference between the two groups in terms of age at offence/admission (OFS mean 36.8 years old, NOFS mean 40.5 years of age, *p=*0.462), their native country being "Switzerland" (OFS 61.3%, NOFS 41.4%, OR=2.24, CI=0.79-6.3, *p=*0.332), their marital status being "single" (OFS 70%, NOFS 44.8%, OR=2.87, CI=0.99-8.37, *p=*0.147) or employment status at the time of the offence/admission (OFS 34.5%, NOFS 12%, OR=3.86, CI=0.93-16.11, *p=*0.065).

**Table 1:** Sociodemographic factors

Variable	Non-offender females with SSD n/N (%)	Offender females with SSD n/N (%)	Odds ratio (OR)	95% Confidence interval (CI)	p value
Age at offence/admission (mean, SD)	40.5 (13.2)	36.8 (10.5)			0.462^1^
Country of birth: Switzerland	12/29 (41.4%)	19/31 (61.3%)	2.24	0.79-6.3	0.332^2^
Single at time of offence/admission	13/29 (44.8%)	21/30 (70%)	2.87	0.99-8.37	0.147^2^
Highest graduation at time of offence/admission: No compulsory school/no graduation	0/22 (0%)	8/29 (27.6%)	1.38	1.1-1.73	*0.031^2^
Unemployment	3/25 (12%)	10/29 (34.5%)	3.86	0.93-16.11	0.065
Homeless at time of offence/admission	0/27 (0%)	7/30 (23.3%)	1.3	1.07-1.59	*0.035^2^
Social Isolation before offence/admission	9/26 (34.6%)	21/29 (72.4%)	4.96	1.58-15.6	*0.026^2^

SD = Standard deviation

### Reported isolation

Secondly, the reported social isolation was significantly higher for OFS (72.4%) than for NOFS (34.6%, OR=4.96, CI=1.58-15.6, *p=*0.026). Social isolation includes few social contacts, missing profound connections or taking part in social activities (see appendix for further information).

### SSD psychopathology

Thirdly, as seen in Table 2, OFS suffered significantly more often from delusions (eq. a false belief that is immune to any sort of evidence or convincing proof, 96.8%) than NOFS (63%, OR=17.65, CI=2.08-149.99, *p=*0.014) were more likely to suffer from self-disorders (51.6%) than NOFS (11.1%, OR=8.53, CI=2.12-34.32, *p=*0.011) at the time of the initial diagnosis (self-disorders hinder the patient from distinguishing between their person/personality and the environment; the term is further explained in the appendix). The two groups were not found to differ significantly in the frequencies of hallucinations (eq. perception without any external input: OFS 64.5%, NOFS 55.6%, OR=1.46, CI=0.51-4.19, *p=*0.652), disorder of drive (eq. manic or depressed symptoms) OFS 64.5%, NOFS 51.9%, OR=1.69, CI=0.59-4.85, *p=*0.519) or negative symptoms (such as diminished thinking, psychomotor movements, drive or emotion; OFS 71%, NOFS 74.1%, OR=0.86, CI=0.27-2.75, *p=*1.000) when they were initially diagnosed. However, cognitive impairment seemed to be more present within the group of NOFS (95.8%) than OFS (67.7%, OR=0.91, CI=0.11-0.78, *p=*0.041; cognitive impairment includes intelligence, fluency in speech, verbal skills and more, see appendix for further details).

**Table 2:** Psychiatric History

Variable	Non-offender females with SSD n/N (%)	Offender females with SSD n/N (%)	Odds ratio (OR)	95% Confidence interval (CI)	*p* value
Delusions at the time of initial diagnosis	17/27 (63%)	30/31 (96.8%)	17.65	2.08-149.99	*0.014^1^
Hallucinations at the time of initial diagnosis	15/27 (55.6%)	20/31 (64.5%)	1.46	0.51-4.19	0.652^1^
Self-disorders at the time of initial diagnosis	3/27 (11.1%)	16/31 (51.6%)	8.53	2.12-34.32	*0.011^1^
Disorders of Drive at the time of initial diagnosis	14/27 (51.9%)	20/31 (64.5%)	1.69	0.59-4.85	0.519^1^
Negative symptoms at the time of initial diagnosis	20/27 (74.1%)	22/31 (71%)	0.86	0.27-2.75	1.000^1^
Cognitive Impairment before offence/ admission	23/24 (95.8%)	21/31 (67.7%)	0.91	0.11-0.78	*0.041^1^
Suicide Attempt ever before offence/admission	7/27 (25.9%)	13/30 (43.3%)	2.19	0.71-6.72	0.418^1^
Endangerment of others present before offence/admission	15/25 (60%)	20/29 (69%)	1.48	0.48-4.55	0.663^1^
Alcohol abuse present in psychiatric history	6/28 (21.4%)	12/29 (41.4%)	2.59	0.81-8.31	0.284^1^
Substance use disorder present in psychiatric history	6/27 (22.2%)	16/31 (51.6%)	0.27	0.09-0.85	*0.039^1^
Personality Disorder present in psychiatric history	5/29 (17.2%)	5/30 (16.7%)	0.96	0.25-3.74	1.000^1^
Other psychiatric comorbidity present in psychiatric history	8/29 (27.6%)	12/30 (40%)	1.75	0.59-5.23	0.533^1^
Outpatient treatment before offence/admission	24/27 (88.9%)	21/30 (70%)	0.292	0.07-1.22	0.218^1^
Inpatient (stationary) treatment before offence/admission	29/29 (100%)	25/30 (83.3%)	1.2	1.02-1.41	0.127^1^
Antipsychotic medication prescribed before offence/admission	28/28 (100%)	22/31 (71%)	1.41	1.13-1.77	*0.022^1^
Antipsychotic medication regularly taken before offence/admission	22/26 (84.6%)	1/22 (4.5%)	0.09	0.00-0.08	*0.000^2^

N is statistically adjusted, no excluding criteria for the offender patients. The non-offender cohort group was balanced after year of evaluation and sex

^1^ p value derived from Mann-Whitney-U-Test

^2^ p value derived from Fisher's exact test and adjusted by the method of Benjamini and Hochberg

* Significant p value

### Internal and external aggression

Fourth, no significant difference was found regarding suicide attempts in their psychiatric history of both groups (OFS 43.3%, NOFS 25.9%, OR=2.19, CI=0.71-6.72, *p=*0.418) or the endangerment of others before the offence/admission (OFS 69%, NOFS 60%, OR=1.48, CI=0.48-4.55, *p=*0.663).

### Comorbidity

Fifth, OFS suffered comorbid substance use disorder significantly more often prior to their admission (51.6%) than NOFS (22.2%, OR=0.27, CI=0.09-0.85, *p=*0.039). The two groups showed no difference in alcohol abuse (OFS 41.4%, NOFS 21.4%, OR=2.59, CI=0.81-8.31, *p=*0.284), personality disorder (OFS 16.7%, NOFS 17.2%, OR=0.96, CI=0.25-3.74, *p=*1.000) or any other psychiatric comorbidity (OFS 40%, NOFS 27.6%, OR=1.75, CI=0.59-5.23, *p=*0.533) prior to or at the time of the offence/admission.

### Treatment

Sixth and last, the two groups did not have any difference in terms of having received treatment (in or outpatient) prior to the admission/offence (outpatient: OFS 70%, NOFS 88.9%, OR=0.292, CI=0.07-1.22, *p=*0.218; inpatient: OFS 83.3%, NOFS 100%, OR=1.2, CI=1.02-1.41, *p=*0.127). However, the NOFS had antipsychotic medication prescribed significantly more often (100%) by a physician/doctor than OFS (71%, OR=1.41, CI=1.13-1.77, *p=*0.022) and the NOFS showed higher medication compliance (84.6%) in comparison to the OFS (4.5%, OR=0.09, CI=0.00-0.08, *p=*0.000).

## Discussion

The main intention of this study was to increase knowledge about the association between criminal behavior and schizophrenia spectrum disorder (SSD) among female patients. Our research showed that OFS were more likely to be socially isolated, showed more positive symptomatology in their medical history and more substance use disorder. OFS were less likely to have completed compulsory school, were less cognitively impaired (please consult appendix for details of assessment) and were notably less compliant if they received antipsychotic medication as compared to non-offender female patients with SSD.

### Contextualization to previous articles

We observed that OFS completed compulsory school less often than NOFS, which contradicts Landgraf et al. 2013, who found no group difference regarding years of school education. Weak associations between any violence and lower levels of education were described for first-episode psychosis patients (Large and Nielssen 2011).

No difference was found regarding unemployment between two groups in our study, whereas Landgraf et al. 2013, found that their female offender patients had a higher unemployment rate than the NOFS.

Homelessness as a risk factor for criminal behavior has been described by previous authors in both female and mixed-sex populations (Landgraf et al. 2013; Ran et al. 2010). Foster et al. 2012, wrote that patients with SSD had a higher risk of homelessness when they had severe psychiatric symptoms, serious impairment in social or school functioning and substance use disorder. Some of these factors are represented by this study’s OFS.

OFS were more likely to suffer delusions and self-disorders at the time of their SSD diagnosis than NOFS. Consulting previous literature, we found a rather consistent association between positive psychotic symptoms, such as delusion, and violent behavior among patients with SSD (during an acute psychotic episode: Hodgins 2022; Wolf et al. 2023), (Ekinci and Ekinci 2013; Swanson et al. 2006; Witt et al. 2013; Wu et al. 2018). Fleischman et al. 2014, quoted literature that suggests schizophrenia to be a stronger risk factor for violent behavior for women than for men (this might because female patients with SSD presented more positive symptoms). However, our study’s OFS did not suffer more from hallucinations than NOFS, which did not fully align with the thesis of positive symptoms being a risk factor for violent behavior. Furthermore, the delusions investigated by our study were presented during the initial diagnosis, not necessarily during the crime.

Our groups showed no difference in negative symptoms at the time of the initial diagnosis. Swanson et al. 2006, stated that negative symptoms could have an alleviating effect on positive symptoms in SSD and that SSD-patients with stronger negative symptoms were associated with having a reduced risk of serious violence.

Cognitive impairment occurred more often amongst NOFS than among OFS, which is controversially discussed in previous literature. For example, Landgraf et al. 2013, found no group difference regarding mental retardation and raised the question: if neurocognitive impairment might serve as an indicator for the development of violent behavior among women. Our results would not support this theory. Slotboom et al. 2017, suggested the possibility that offender patients (OP) engaged with higher motivation to perform well in cognitive tests and thus scored higher. However, Slotbloom also found that OP might have superior attention than healthy community controls, but their study did not focus exclusively on individuals with SSD. In contrast to our findings, Reinharth et al. 2014, found evidence suggesting impairment in global cognitive ability to relate to an increase in the likelihood of aggression. Ahmed et al. 2018, stated that cognitive deficits increase the risk of impulsive aggressive behavior in patients with schizophrenia. One possible explanation for this discrepancy in results could lie in the mixed-sex populations of both Ahmed et al. 2018 and Reinharth et al. 2014, which would suggest that cognitive impairment might play a larger role in the development of violence in men than in women. Further studies are needed to support this hypothesis.

The SSD population had an increased rate of substance use disorder (Kirchebner et al. 2023; Volkow 2009) compared with the general population. Substance use disorder is a known risk factor for criminal behavior among the general population. An association between substance use disorder and criminal behavior among SSD patients has also been identified by Fazel et al. 2009, who wrote that the risk for violence increases about four times, if the patients with SSD also suffered from substance use disorder, than if they had not. Substance abuse is seen as the most considerable predictor of violent behavior for people with mental health disorders. (Barlati et al. 2022) and Duke et al. 2018, wrote that the association between drugs and violence appears to be even stronger for individuals with psychotic illness. We confirmed this observation in female offender patients, as did Landgraf et al. 2013.

We found no significant difference in the frequency of alcohol abuse between OFS and NOFS, however, our study showed that only 21.4% of the NOFS versus 41.4% of the OFS had a history of alcohol abuse. Landgraf et al. 2013 had significant findings of higher rates of "mental and behavioral disorders due to the use of alcohol" for OFS and they stated that "females being diagnosed with schizophrenia is associated with severe comorbid substance or alcohol abuse".

In order for us to agree with their statement, we would need to generate more knowledge about the female offender SSD population, which would require a greater number of participants in order for us to properly compare them to male offender patients suffering from SSD.

All of the NOFS had antipsychotic medication prescribed before admission to the reported hospitalization, unlike OFS. This observation was crucial, as atypical neuroleptics might decrease the rate of substance use disorder (J. Swanson et al. 2007) and antipsychotic medication is considered to reduce positive symptoms (Hodgins 2022; Swanson et al. 2004) and thus was linked to reduce criminal behavior and/or violence/aggression (Fazel et al. 2014; Krakowski et al. 2021; Strassnig et al. 2020; Swanson et al. 2004, 2006; Wu et al. 2018) in patients with SSD.

Regular intake of antipsychotic medication was notably significantly lower in the OFS group, among which, only one woman was compliant with her prescribed medication before committing the crime. Witt et al. 2013, found that in psychosis violence risk was moderately associated with medication non-adherence.

Unexpectedly there was no difference observed between OFS and NOFS regarding suicide attempts before the crime/admission. This contradicted the finding by Landgraf et al. 2013. Lee et al. 2012, which found that external violence has predictable value for suicide attempt and Hor and Taylor 2010, list delusions, substance use disorderand lack of compliance, among others, as risk factors for suicide.

There was unexpectedly no difference found regarding endangerment of others before admission/offence. This was incongruent with the finding by Machetanz et al. 2023, who had a much higher rate among their mixed-sex OP (they studied the same female patients as included in this study).

We could not find any difference regarding personality disorder (neither did Landgraf et al. 2013) or other psychiatric comorbidity in psychiatric history among the two groups.

Last but not least, we observed no difference regarding previous regular psychiatric/ psychotherapeutic in or outpatient treatment between the two groups. We would have expected to receive significantly higher results for NOFS, as we hypothesized that therapy could help prevent them from committing offences, as Kirchebner et al. 2023, suggested likewise for their mixed groups. As we stated above NOFS were more likely to have antipsychotics prescribed and they showed better compliance. Landgraf et al. 2013 showed more previous inpatient hospitalizations among the OFS group. Explanations could be the measuring of previous treatment being qualitative and not quantitative – maybe the OFS had not attended therapy often enough to be diagnosed and medicated correctly. Unfortunately we had no information about the reasons for previous hospitalizations, due to the retrospective design, which made it impossible to clarify whether medication prescription and previous hospitalizations were correlated.

Finally, comparing our female population to a male population is definitely not sufficient. The shortage of studies specifically focusing on female offender patients with a SSD diagnosis shows how important it is to generate basic knowledge in this field of research.

### Strengths and weaknesses

This study has limitations. As the percentage of females with SSD in forensic psychiatry is small, the total number of participants in our cohorts was limited. This indicates that the statistical power was not as strong as it would have been, had we used a larger sample. Despite the small sample, the results were notable: Using the Benjamini Hochberg method in addition to the Fisher’s exact test helped decrease the false discovery rate, as it helped to avoid type 1 errors. Our results thus included fewer false positive outcomes and were valid. For further research on this population, a greater number of cases needs to be included. This would allow researchers to carry out subgroup analyses and to differentiate between the various comorbidities (for example substance use disorder).

As we had broad inclusion criteria, the sample might not be homogenous and thus definitive conclusions are difficult to draw. We could not deliver an explanation for the malcompliance with antipsychotic medication prescription in the female offender cohort. This begged the question as to why some of the OFS did not receive antipsychotic treatment. A possible explanation might be a lack of insight into the need for treatment, dissimulation (eq. the patients understated their suffering and symptoms towards their therapists and might not have provided all of the required information) or anosognosia (eq. the patients did not recognize the severity of their illness). The question remains if these female patients were identified as mentally ill before committing their crime. It was notable that the variables obtained from the data in this study were taken from patient questionnaires filled out by their therapists. Thus, the data had limits regarding its verifiability and correctness. It was difficult to differentiate between what was reported by the therapist about what happened versus the patient’s point of view. It should be understood that this study did not include all of the possible SSD-symptoms, which could occur during or before the illness.

Another inevitable limitation was that the forensic sample was not investigated in the psychiatric hospital but at their entry in the judicial system, when they were charged with their crime. Consequently, OFS and NOFS were not analyzed and questioned at the same point in the therapeutical procedure. The biggest statistical weakness was the retrospective design. With gender medicine emerging and putting an emphasis on the importance of gender differences, this study helps cotntribute to a wider knowledge about women’s disease and their illness behavior. This study matters because comparing only female offender patients with SSD to female non-offender patients with SSD is rare and thus current knowledge is limited.

### Future research implication

We encourage further research on more SSD specific symptoms, in order to find more conclusive information about the association between positive SSD symptoms and violence among female patients. It is also important to measure positive SSD symptoms at the time of the crime. Furthermore, it would be useful to investigate the association between cognitive impairment and criminal behavior, and to provide fewer conflicting results. As we did not find any difference regarding endangerment of others before admission, it would be interesting to clarify, if this is typical for female SSD offender patients. Further research could examine the endangerment of others during hospitalization to see if it differs from behavior before the admission and to further investigate suicidal behavior among SSD female patients. It would be important to know if in or outpatient treatment has preventative effects on the amount of crimes committed among female SSD patients.

Why does schizophrenia seem to affect the association with violent behavior more in female patients than in male patients? It would be interesting to investigate female patients’ behavioral changes after their first psychotic episode in comparison to their behavior before the illness. This could reveal an explanation of why female patients SSD diagnosis seem to affect violent behavior more than in men. This study’s results should be applicable to other European countries with similar judicial correctional facilities that are forensic psychiatric institutions.

### Clinical implication

The clinical implication could be to look at OFS’s needs differently and focus more on the effective treatment practices for this specific group. Women might make up a special offender cohort, which might need their own therapy concepts and facilities. As seen in male patients, it is necessary to target substance use disorder in female patients with SSD, in order to reduce criminal behavior. We emphasize the importance of the regular intake of neuroleptic medication (compliance), which could be simplified through long-acting injectable antipsychotics or, in the future, in the form of antipsychotic implants previous authors have discussed (Rabin et al. 2008; Siegel 2002; Utomo et al. 2022) to help reduce positive SSD symptoms and associated criminal behavior. An early detection of the illness is crucial in order to provide support for these individuals, and to prevent them from being stigmatized and living on the edge of society. An early integration in the medical support system is necessary in order to treat SSD-symptoms correctly and efficiently. We propose that other researchers differentiate between women and men in their future research. Otherwise, the risk remains that either group could receive ineffective treatment. We advise researchers to share their data and findings, in order to create larger cohort groups and thus to assemble stronger evidence. Further research could also focus on holistic inclusion of risk factors and pathology in female offender patients suffering from SSD (OFS).

## Conclusion

In conclusion: offender female patients with schizophrenia spectrum disorder (OFS) can be differentiated sociodemographically, clinically and in treatment adherence from non-offender female patients (NOFS). We found that OFS present themselves as a unique sample in the patient group suffering from SSD, who require special attention regarding their pre-criminal behavior, screening, medical compliance, and appropriate therapy approaches.

## Data Availability

The dataset generated and analyzed during the current study is available from the corresponding author upon reasonable request. A detailed list of all our variables (including definitions and references) is available under the following link: https://www.researchgate.net/publication/363044110_Coding_protocol_Pathways_into_delinquency_in_offenders_suffering_from_schizophrenia_spectrum_disorders.

## Appendix

1. Abbreviations

- SSD: schizophrenia spectrum disorder (includes the subtypes hebephrenic and paranoid Schizophrenia as well as acute psychotic and schizoaffective disorder)
- OFS: offender female patients with SSD
- NOFS: non-offender female patients with SSD
- NOP: non-offender patients (including both sexes)
- OP: offender patients (including both sexes)

2. Definitions for chosen variables in Table 1 and Table 2

- Age at admission: SD1: Age (in years and months) at the date of admission to the referenced forensic hospital.
- Country of birth: Switzerland: SD3: Country of birth as indicated in the file.
- Unemployment: The variable was dichotomized and defined as "yes" if, at the time of admission, the patient had been without any job during > 50% of working age (counted from the 15th birthday or the graduation date, respectively). The term ‘job’ includes temporary and part-time employment.
- Single at time of offence/admission: SD5b: Marital Status at the time of the investigated offence/admission: Single? Yes, if he/she was unmarried at the time of the investigated offence/admission.
- Highest graduation at time of offence/admission: No compulsory school/no graduation: SD7a: Yes, if he/she had not completed primary or (lower) secondary school education (school period from about age 6 to about age 16) at the time of the investigated offence/admission (European Commission/EACEA/Eurydice 2018).
- Homeless at time of offence/admission: SD6g: Living situation at the time of the investigated offence/admission: Homeless? Yes, if, at the time of the investigated offence/admission, he/she lived in a place which was below the minimum housing standard AND had no access to an adequate dwelling (Amore et al. 2011).
- Social Isolation before offence/admission: S9i: Stressors in the recent past: Isolation? Yes, if any report in the file states that he/she had suffered from social isolation and/or its consequences for any period of time before the investigated offence/admission. Note: Signs of social isolation include "small social networks, infrequent social contacts, absence of confidante connections, living alone, and lack of participation in social activities" (Tanskanen and Anttila 2016, p. 2042).
- Delusions at the time of initial diagnosis: PH3a: Delusions at the time of the initial diagnosis? Yes, if he/she (had) experienced any type of delusion (persecutory delusions, delusions of grandeur, etc.) (Arbeitsgemeinschaft für Methodik und Dokumentation in der Psychiatrie 2023) at the time of the initial diagnosis? Note: ‘Delusion’ is defined as "a false belief based on incorrect inference about external reality that is firmly sustained despite what almost everybody else believes and despite what constitutes incontrovertible and obvious proof or evidence to the contrary. The belief is not one ordinarily accepted by other members of the person’s culture or subculture (e.g. it is not an article of religious faith)" (American Psychiatric Association 2013)
- Hallucinations at the time of initial diagnosis: PH4a: Hallucinations at the time of the initial diagnosis? Yes, if he/she (had) experienced any visual, auditory, olfactory, tactile and/or gustatory hallucinations (Arbeitsgemeinschaft für Methodik und Dokumentation in der Psychiatrie 2023) at the time of the initial diagnosis?
- Self-disorders at the time of initial diagnosis: PH5a: Penetrability of the own ego at the time of the initial diagnosis? Yes, if he/she (had) experienced any of the following at the time of the initial diagnosis: thought insertion, thought withdrawal, thought broadcasting, thought echo depersonalisation, derealisation and/or delusions of control (Arbeitsgemeinschaft für Methodik und Dokumentation in der Psychiatrie 2023).
- Disorders of Drive at the time of initial diagnosis: PH6a: Disorders of affect or drive at the time of the initial diagnosis? Yes, if he/she (had) experienced any mania- and/or depression-like symptoms with the consequence of any type of functional impairment (distress and/or disability) (American Psychiatric Association 2013; Üstün and Kennedy 2009) at the time of the initial diagnosis.
- Negative symptoms at the time of initial diagnosis: PH7a: Negative symptoms at the time of the initial diagnosis? Yes, if he/she (had) experienced any types of negative symptoms which are listed in the positive and negative syndrome scale (PANSS) at the time of the initial diagnosis (Kay et al. 1987) which cannot be better explained by other causes (such as, for example, another mental illness).
- Cognitive impairment before offence/admission: N2: Did the patient show a cognitive deficit? Yes, if any report in the file states that he/she had shown deficits in any of the domains "general intelligence" (N1b), "attention", "verbal memory", "verbal fluency", "verbal learning and memory" or "executive functioning" (adopted from Bowie and Harvey 2006, p.532 - 533) for a period of at least 1 year before offence/admission to the referenced forensic hospital.
- Suicide attempt ever before offence/admission: PH10a: Did the patient ever attempt suicide? Yes, if he/she had harmed himself/herself intentionally with suicidal intent at any one time before the investigated offence/admission
- Endangerment of others present before offence/admission: PH11a: Did the patient ever endanger others? Yes, if, at any one time before the investigated offence/admission, he/she had deliberately or negligently put one or more other person(s) at any type of risk (e.g., by the exertion of violence), which entailed the potential or actual consequence of a substantial mental and/or physical impairment of the corresponding person(s).
- Alcohol abuse present in psychiatric history: PH13: Is there or was there any alcohol abuse? Yes, if he/she (had) had an alcohol consumption pattern corresponding to the ICD-10 diagnosis "Harmful use (F10.1)" or "Dependence syndrome (F10.2x)" at the time of or at any one time before the investigated offence/admission (World Health Organization 2004).
- Substance use disorder present in psychiatric history: PH14a: Is there or was there any substance use disorder? Yes, if he/she did not have and had not had a substance consumption pattern corresponding to the ICD-10 diagnosis "Harmful use (F1x.1)" or "Dependence syndrome (F1x.2x)" at the time of and at any one time before the investigated offence/admission (World Health Organization 2004). Note: In this item, the term ‘substance’ refers to illicit drugs and prescription medications but not to alcohol.
- Personality Disorder present in psychiatric history: PH15a: Presence of a personality disorder (PD)? Yes, if he/she was diagnosed with any personality disorder listed in the ICD-10 (F60.x, F61) and/or the DSM-V at the time of or at any time before the investigated offence/admission (American Psychiatric Association 2013; World Health Organization 2004).
- Other psychiatric comorbidity present in psychiatric history: PH17a: Any other psychiatric comorbidity? Yes, if he/she was diagnosed with any mental illness listed in the ICD-10 or DSM-5 at the time of the investigated offence/admission except for… (American Psychiatric Association 2013; World Health Organization 2004).
  - schizophrenia spectrum disorders (ICD-10: F2x.x) and related disorders in the DSM-5
  - personality disorders (ICD-10: F60.x, F61) and related disorders in the DSM-5
  - harmful use (ICD-10: F1x.1), dependence syndromes (F1x.2x) and other substance related disorders in the DSM-5
- Outpatient treatment before offence/admission: PH18a: Any psychiatric outpatient treatment(s) before the investigated offence/admission? Yes, if he/she had visited a mental health care provider (psychologist and/or psychiatrist) as an outpatient at any time before the investigated offence/admission, regardless of the duration of said treatment.
- Inpatient (stationary) treatment before offence/admission: PH19a: Any psychiatric inpatient treatment(s) before the investigated offence/admission? Yes, if he/she had been an inpatient and/or a semi-inpatient in a mental health care institution at any time before the investigated offence/admission, regardless of the duration of said treatment.
- Antipsychotic medication prescribed before current hospitalization: PH23a: Had the patient received any neuroleptic medication before the investigated offence/admission? Yes, if he/she had been prescribed any antipsychotic medication at any time before the investigated offence/admission.
- Antipsychotic medication regularly taken before current hospitalization: Ph23p: If ‘yes’ in PH23a: Had the neuroleptic medication been consumed regularly before the investigated offence/admission? Yes, if he/she, mental health professionals and trusted private persons (e.g., close family members) had not reported/documented a lack of compliance/adherence to any antipsychotic medications at any time before the investigated offence/admission AND if mental health professionals and trusted private persons (e.g., close family members) had not had reasonable grounds for suspecting that the patient lacked medication compliance/adherence to any antipsychotic medications at any time before the investigated offence/admission.

Source: "Coding protocol by Kirchebner et al.: Offender patients with schizophrenia spectrum disorder (SSD) – Database"

"Note: All items reflect the contents of the patients’ files. Due to the retrospective design, a direct evaluation with the patient through the investigators was not possible. "Offence" refers to the offence leading to the referenced forensic hospitalization."

## Declarations

Conceptualization: Lynn Jacobshagen (LJ), Lena Machetanz (LM), Johannes Kirchebner (JK); Methodology: LM, JK; Formal analysis and investigation: JK; Writing – original draft preparation: LJ, LM; Writing – review and editing: LJ, LM, JK; Supervision: LM, JK. Special thanks to Myva Newman.
